# Fibrosis: Shared Lessons From the Lens and Cornea

**DOI:** 10.1002/ar.24088

**Published:** 2019-03-18

**Authors:** A. Sue Menko, Janice L. Walker, Mary Ann Stepp

**Affiliations:** ^1^ Department of Pathology, Anatomy, and Cell Biology Thomas Jefferson University Philadelphia Pennsylvania; ^2^ Department of Anatomy and Cell Biology George Washington University Washington District of Columbia

**Keywords:** fibrosis, cornea, lens, fibrocyte, myofibroblast

## Abstract

Regenerative repair in response to wounding involves cell proliferation and migration. This is followed by the reestablishment of cell structure and organization and a dynamic process of remodeling and restoration of the injured cells' extracellular matrix microenvironment and the integration of the newly synthesized matrix into the surrounding tissue. Fibrosis in the lungs, liver, and heart can lead to loss of life and in the eye to loss of vision. Learning to control fibrosis and restore normal tissue function after injury repair remains a goal of research in this area. Here we use knowledge gained using the lens and the cornea to provide insight into how fibrosis develops and clues to how it can be controlled. The lens and cornea are less complex than other tissues that develop life‐threatening fibrosis, but they are well characterized and research using them as model systems to study fibrosis is leading toward an improved understanding of fibrosis. Here we summarize the current state of the literature and how it is leading to promising new treatments. Anat Rec, 2019. © 2019 The Authors. *The Anatomical Record* published by Wiley Periodicals, Inc. on behalf of American Association of Anatomists.

## INTRODUCTION

The ability to repair damaged body parts evolved early during evolution and has been studied over the years in all multicellular organisms. Despite the many contributions that have informed us about the mechanisms of regenerative repair, where tissue and/or organ functionality is fully restored after injury, and non‐regenerative repair that results in fibrosis, there is limited knowledge about how an environment created to ensure regenerative repair instead becomes one that promotes fibrosis. A major goal in biomedical science is to enhance the tissue regeneration process and prevent fibrosis. While all injured tissues are tasked with maintaining their function while they carry out the repair process, this is a particular challenge in the eye, where light transmission from the surface of the cornea through the lens to the retina must be retained during and after wound repair is complete.

Regenerative repair in response to wounding involves cell proliferation and migration. This is followed by the reestablishment of cell structure and organization via a dynamic process of remodeling and restoration of the injured cells' extracellular matrix microenvironment as the regenerated region is integrated into surrounding tissues (Bonnans et al., 2014). This involves expression of matrix proteins associated with development, suggesting that the same matrix microenvironment that supports tissue formation is also essential to the tissue regeneration process. Later, the remodeling of that environment and *de novo* synthesis of other matrix elements recreates a matrix environment that replicates that present before injury (Bonnans et al., 2014). An imbalance in injury induced matrix production and/or defects in remodeling often results in sustained and progressive fibrosis in and around sites of injury and impairs the regeneration process (Bonnans et al., 2014). A fibrotic outcome is the major limiting factor in regenerative repair of a wound and leads to a loss of tissue function (Walraven and Hinz, 2018).

The microenvironment created for the normal wound healing process involves many of the same matrix elements that promote fibrosis, including fibronectin, tenascin C, and collagen I. Early in the repair process, fibronectin EDA and tenascin C form a provisional matrix that supports cell proliferation and migration, while serum‐derived fibrin is central to forming a blood clot in the wound bed (Rousselle et al., 2018). A collagen I‐rich matrix is then assembled that strengthens the wound site (Rousselle et al., 2018). In the skin and cornea this matrix is referred to as a scar that in the cornea can result in hazing, which persists if the wound repair matrix environment is not resolved (Wilson et al., 2017). Following wound closure, the matrix associated with wound repair is remodeled (Bonnans et al., 2014). Macrophages present at the wound site secrete matrix metalloproteinases (MMPs) that cleave collagen and phagocytose the resultant collagen fragments (Madsen et al., 2013). The resolution of the matrix environment assembled for repair distinguishes the normal, regenerative wound healing process from fibrotic repair, characterized by the production of an excessive collagen I/fibronectin‐rich matrix environment that is stabilized by collagen cross‐linking enzymes like lysyl oxidase (Li et al., 2018). A fibrotic matrix environment is difficult to resolve, destroys tissue architecture, and impairs tissue and organs from carrying out their normal function.

Among the cells that have been identified as producers of collagen I and other matrix proteins in both wound healing and fibrosis are fibroblasts, fibrocytes, and myofibroblasts (Reilkoff et al., 2011; Peng and Herzog, 2012). Fibroblasts within the connective tissue adjacent to the site of injury become activated. Fibrocytes, bone marrow mesenchymal‐derived CD45+/collagen I+ cells, are recruited to the wound to modulate the repair process (Herrera et al., 2018). The myofibroblasts that emerge following wounding express α‐smooth muscle actin (αSMA), which is organized into stress fibers. Myofibroblasts can be derived from a number of different mesenchymal cell types including immune cells (fibrocytes and macrophages), pericytes, Schwann cells, and fibroblasts (McAnulty, 2007; Kramann et al., 2013).

Fibrosis can affect almost every tissue in the body. In pulmonary fibrosis, thick scar formation compromises the area around the air sacs (alveoli) impairing the passage of oxygen to the blood and leads to a progressive loss of lung function over time. Scarring of the skin following wounding or surgery can be unattractive, and excessive matrix production, as in the formation of keloids, disfiguring. Post‐surgery fibrosis causes internal adhesions that result in the failure of many surgical procedures. In the eye, fibrotic outcomes lead to loss of vision including corneal fibrosis (Wilson, 2012), posterior capsule opacification (Apple et al., 1992), idiopathic epiretinal membrane (Bu et al., 2014), and proliferative vitreoretinopathy (Pennock et al., 2014). While fibrosis is one of the most extensively covered research topics in biomedical science with active research programs that cover all of the tissues in the body, there are currently no treatments that will stop or reverse its progression. Ideally, the goal for regenerative medicine is to induce tissues to reactivate repair programs to restore function. Here we will use examples from the anterior aspect of the eye, lens, and cornea, to discuss the advances made in our understanding of pathological fibrosis, its mechanism of action, its relationship to regenerative repair and future directions for research.

## LESSONS LEARNED FROM THE STUDY OF THE LENS

### Immune Surveillance and Fibrosis in the Dysgenic Lens

In response to injury or dysgenesis, tissues become rapidly populated by immune cells that perform a number of distinct functions aimed at insuring regenerative repair (Gong et al., 1994; Pellicoro et al., 2014; Rogers et al., 2014). Resident macrophages and dendritic cells, among the earliest immune cells to populate an injury site, recruit circulating monocytes from the vasculature. Immune cells function at the site of injury in both transient inflammatory responses and to mediate the repair process itself. Included among the cell types called to the site of injury are bone marrow mesenchymal‐derived fibrocytes, characterized by their co‐expression of the immune cell protein CD45 and collagen I (Herrera et al., 2018). Fibrocytes, together with macrophages, play a role in matrix production and remodeling during the repair process. Fibrocytes secrete growth factors in addition to collagen I. Macrophages migrate to the wound edge where they function as leader cells. Leader cells regulate collective migration of epithelial cell sheets after injury (Omelchenko et al., 2003; Chapnick and Liu, 2014; Yamaguchi et al., 2015) and release factors required for repair including regulatory cytokines and MMPs. These MMPs are essential to remodeling the provisional extracellular matrix environment produced following injury to promote regenerative repair (Bonnans et al., 2014).

The myofibroblasts that appear post‐wounding and become a causative factor in fibrosis arise from the immune and bone marrow mesenchymal cells that populate wounded and dysgenic tissues (McAnulty, 2007; Kramann et al., 2013). The differentiation of these repair cells to a myofibroblast phenotype is identified by their expression of αSMA. The organization of αSMA into stress fibers confers myofibroblasts with properties associated with movement and contraction. While for many years the sources of myofibroblasts in wounded or dysgenic tissues were unclear, current literature now provides strong evidence that the progenitors of these myofibroblasts have a mesenchymal origin. Fibrocytes and macrophages have been identified as the principle sources of fibrotic disease–associated myofibroblasts (McAnulty, 2007; Kramann et al., 2013). The accumulation, organization, and stabilization of matrix proteins like fibronectin and collagen I produced to promote the wound repair create an environment with increased stiffness that promotes fibrosis (Miller, 2017). It is in these altered microenvironments that immune cells like macrophages and fibrocytes receive mechanotransduction signals that induce their differentiation to myofibroblasts. The proliferation of myofibroblasts and their production of collagen I expands and extends the fibrotic environment (Tomasek et al., 2002; Walraven and Hinz, 2018), which then interfere with the repair process and the normal function of the tissue and is difficult to resolve.

Because the adult lens is avascular and was considered an immune privileged tissue, the potential role of immune cells in fibrosis in the lens had not been considered. While macrophages (F4/80+ cells) were shown to populate the lens early in development when the lens vesicle is still open (Nishitani and Sasaki, 2006), and the lens still has access to a vasculature, the fate of these cells and the potential that there might be a requirement for immune cells in the adult lens was not pursued. However, it is now clear that, in the lens, immune privilege is really immune quiescence (Logan et al., 2017a). The lens‐specific immune surveillance mechanism provides it with an extrinsic source of immune cells in response to dysgenesis or injury. The immune cells recruited to the lens for repair were likely to play a role in fibrosis, as they do in other tissues including another avascular organ—the cornea. The discovery that there is a unique mechanism to deliver immune cells to the adult, avascular lens was made in studies with the N‐cadΔlens mouse (Logan et al., 2017a). The targeted loss of N‐cadherin in the lens, a molecule important to cell adhesion, migration, and the regulation of cytoskeletal organization, results in significant lens dysgenesis because of the resulting impairment of the elongation and migration of differentiating lens fiber cells (Logan et al., 2017b).

The immune response activated in response to lens dysgenesis in adult N‐cad^Δlens^ mice includes recruitment of macrophages, leukocytes, B cells, and T cells to the lens, with CD68+ macrophages the first responders, followed by B and T cells (Logan et al., 2017a). The populating of dysgenic lenses by immune cells occurs in the presence of an intact lens capsule and the absence of a lens‐associated vasculature (Logan et al., 2017a). The likely path for these immune cells to travel to the lens was identified as the ciliary zonules that connect the lens to the vasculature‐rich ciliary body.

Studies of the ciliary zonule proteome reveal that among the many molecules associated with these fibrillin‐based structures are matrix proteins that can facilitate immune cell movement (De Maria et al., 2017). One is MAGP1, which links the active form of transforming growth factor β (TGFβ) to fibrillin (Craft et al., 2014). TGFβ signaling has been linked to the promotion of fibrosis and cell migration (Kuonen et al., 2018). Co‐immunolabeling along the ciliary zonules for LYVE‐1, a hyaluronan receptor expressed on the surface of both immune cells and lymphatic endothelial cells, with MAGP1 that increases significantly in N‐cad^Δlens^ mice supports the role of the zonules in the movement of immune cells from the ciliary body to the lens (Logan et al., 2017a). LYVE‐1+ nonendothelial cells have been localized to many regions of the normal mouse eye including the ciliary body and limbus (Xu et al., 2007). These LYVE‐1 cells include many subsets of immune cell types including leukocytes and macrophages and were shown to have a bone marrow mesenchymal origin (Xu et al., 2007). As a cell surface receptor that binds to a matrix ligand, LYVE‐1 can be cleaved and remain linked to the matrix as cells migrate (Nishida‐Fukuda et al., 2016). The increase in labeling for LYVE‐1 along the zonules that occurs in response to lens dysgenesis in N‐cad^Δlens^ mice suggest that it represents a LYVE‐1 fragment left behind by immune cells migrating along the ciliary zonules (Logan et al., 2017a).

The lenses of adult N‐cadΔlens mice develop cataract‐like opacities that are associated with the appearance of α‐SMA+ myofibroblasts and the accumulation of a collagen I matrix, the principal features of fibrogenesis (Logan et al., 2017a). The progenitors of these myofibroblasts were CD45+/β2 integrin+ immune cells that had been recruited to these dysgenic lenses (Logan et al., 2017a). This discovery of a lineage link between immune cells and myofibroblasts that populate dysgenic lenses suggests that other types of fibrogenic cataracts could also be an outcome of the recruitment of immune cells to the lens, both in the case of lens dysgenesis and following wounding or trauma. One such example is anterior subcapsular cataracts (ASCs), a fibrotic cataract characterized by the accumulation of a collagen I matrix that is populated by α‐SMA+ myofibroblasts just under the anterior lens capsule (Xiao et al., 2015). The fact that the principal cause of ASC is ocular trauma resulting in an inflammatory response in the aqueous humor, the fluid located between the cornea and the anterior surface of the lens (Wormstone and Wride, 2011), it seems likely the progenitors of the myofibroblasts associated with ASC are also immune cells recruited to these lenses by cytokine released in response to the ocular trauma.

It is also important to consider whether immune cells could also be the cause of the high level of cataractogenesis in diseases associated with inflammation in the eye. Type 2 diabetes is an autoimmune disease; immunomodulatory strategies are being employed to lower blood glucose levels and reduce the severity and prevalence of complications of disease (Donath and Shoelson, 2011). Major complications of diabetes make it a leading cause of blindness in adults (Skarbez et al., 2010). In the lens, diabetes leads to the development of cataract and poor outcomes of cataract surgery, including posterior capsule opacification (PCO). Similar negative outcomes for the lens are reported for uveitis (Ram et al., 2010; Llop and Papaliodis, 2018). Diabetes and Sjogren's Syndrome, another autoimmune disease, also disrupt corneal homeostasis. The finding that immune surveillance of the lens is induced in response to lens dysgenesis, and that the immune cells recruited across the intact lens capsule have the potential to differentiate to fibrotic disease‐causing myofibroblasts associated with cataract suggest that we need to rethink how cataracts form, especially in diseases like diabetes and uveitis.

Immune cells could be the source of myofibroblasts associated with fibrotic PCO. Studies demonstrate that the myofibroblasts that appear following cataract surgery have a mesenchymal origin (Gerhart et al., 2014; Walker et al., 2018). Resident immune cells like macrophages, as well as fibrocytes and immune cells recruited to the cataract surgery wound site, are all potential sources of myofibroblasts that are linked to fibrotic PCO. The high level of PCO in patients with diabetes or uveitis further emphasizes the need to understand the links between immune cells and fibrotic conditions of the lens. Although the lens is exposed to immune cells traveling from the ciliary body on the ciliary zonules or from inflammatory reactions that involve the aqueous humor, these cells have rarely been considered as causes of fibrotic‐related conditions of the lens. New evidence in the eye, together with substantial literature regarding the close connection between diseases and pathologies that lead to inflammation of the eye, point to the importance of studying the role of immune cells in development of pathologies of the lens.

### Regenerative Versus Fibrotic Repair Post‐Cataract Surgery

In cataract surgery, the opacified fiber cell mass is removed from within the lens capsule, the extracellular matrix that surrounds the lens, within which an intraocular lens (IOL) is inserted to restore vision. Lens epithelial cells that line the inner aspects of the anterior and equatorial surfaces of the capsule remain attached to the capsule post‐cataract surgery. The fiber cells are attached through their basal surfaces to the posterior aspect of the lens capsule; their removal creates a wound edge where they were in contact with the lens epithelium. In response to cataract surgery, a wound healing process is initiated in the lens epithelium to promote repair of the denuded posterior capsule (Wormstone et al., 2009). As is typical of wound repair, these epithelial cells proliferate and migrate across the wounded region of the lens basement membrane. A normal, polarized epithelium is regenerated along the posterior capsule that preserves the transparency of the lens. In 20%–40% of adult cataract surgeries, the wound repair process instead leads to a fibrotic outcome referred to as secondary cataract or PCO, which interferes with light passage to the retina, impairing vision (Nibourg et al., 2015). In pediatric cataract patients under the age of two the incidence of fibrotic repair is as high as 100% (Vasavada et al., 2011). Fibrotic PCO typically appears within a few months to a few years following cataract surgery. There are other forms of PCO that result from the differentiation of lens epithelial cells to fiber cells, which do not involve fibrosis (Wormstone et al., 2009). The aberrant repair process of fibrotic PCO post‐cataract surgery (which from here we will just refer to as PCO) involves mechanisms that are typical of fibrotic disease in other tissues, characterized by the accumulation of matrix proteins such as fibronectin and collagen I, and the emergence of α‐SMA+ myofibroblasts. What triggers an aberrant response to cataract surgery to cause PCO is not clear. Lens cataract surgery is an ideal model for understanding regenerative repair mechanisms and how the wound repair process can become one that results in fibrosis.

Many lens models have been developed to study PCO (Liu et al., 1996; West‐Mays et al., 2010; Wormstone and Eldred, 2016; Boswell et al., 2017). The classic model is the human capsular bag cultures, which have been used to study wound repair and fibrosis (Liu et al., 1996). Based on this model an *ex vivo* mock cataract surgery model was created using embryonic chick lenses, which have the advantage of providing unlimited numbers of age‐matched wounded explants (Walker et al., 2007; Menko et al., 2014a; Walker et al., 2015). Following hydroelution of fiber cells cuts are made in the anterior capsular bag to flatten the explant on a culture substrate. This makes it possible to study concurrently the mechanisms of regenerative repair during wound closure in the endogenous environment of the posterior lens capsule and the mechanisms of fibrosis, an outcome that is induced when the cells migrate from the injured lens capsule onto the surrounding tissue culture platform (Menko et al., 2014a; Menko et al., 2014b; Walker et al., 2018). The microenvironment of the culture platform that these cells encounter can be predetermined, making it possible to examine the impact of substrates with defined stiffness consistent with promoting fibrosis (Walker et al., 2018).

**Figure 1:**
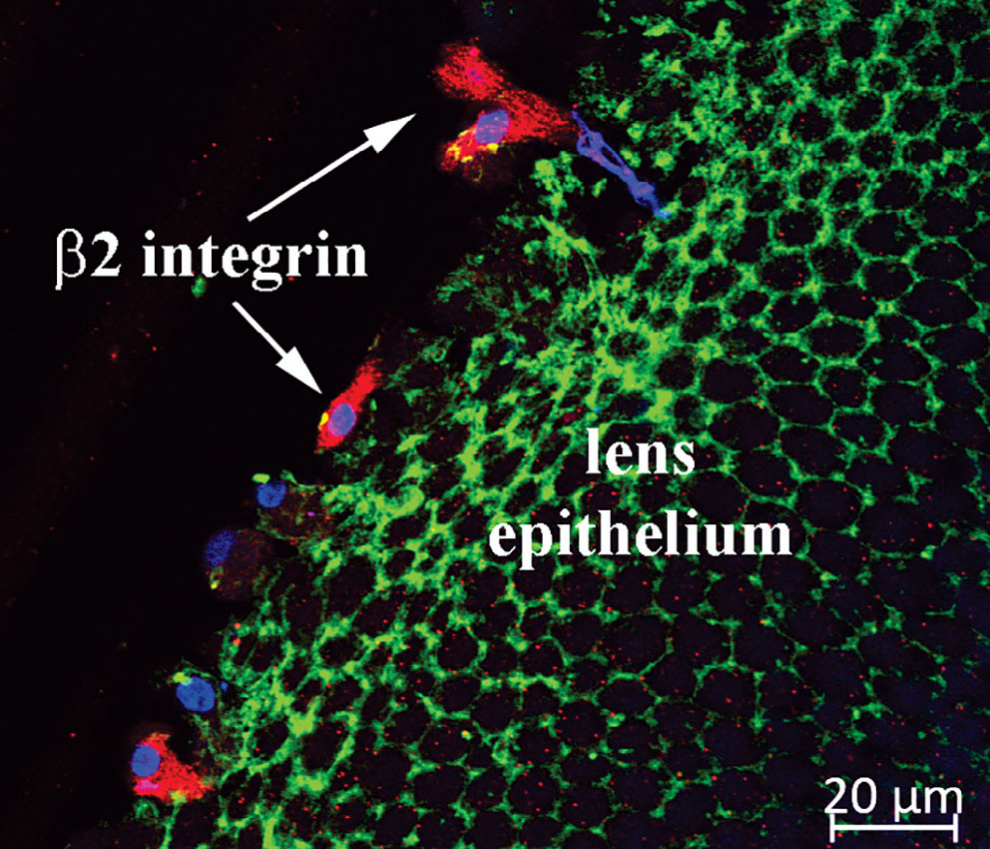
Immune cells go to the wound edge. *Ex vivo* mock cataract surgery wounding was performed on E15 chicken embryo lenses and the explants fixed with 3.7% formaldehyde shortly post‐injury. Explants were imaged by confocal microscopy using Zeiss LSM510 microscope following immunolabeling with an antibody to β2 integrin (Santa Cruz), which recognizes a surface protein expressed by immune cells, and a fluorescent‐conjugated secondary antibody (Jackson Immunoresearch) (red). Lens epithelial cells were detected by co‐labeling for F‐actin (fluorescent‐conjugated phalloidin [Molecular Probes], green). Nuclei were labeled with TOPRO‐3 (Molecular Probes) (blue). Immune cells (β2 integrin+) rapidly locate to the wound edge of the lens epithelium in response to cataract surgery wounding.

In wound repair, the cell types associated with producing collagen I and other matrix proteins in the wound bed have a mesenchymal origin. In fibrosis, mesenchymal cell types including fibrocytes, macrophages, pericytes, Schwann cells, and fibroblasts, which are activated by wounding, and many of which are now believed to be the progenitors of myofibroblasts (McAnulty, 2007; Kramann et al., 2013). Studies of chick, rat, and human cataract surgery identified the presence of an innate population of vimentin‐rich mesenchymal cells in the lens (Walker et al., 2010; Gerhart et al., 2014). In the chick model, it was shown that these mesenchymal cells are rapid responders to wounding, that they express the receptor CD44, and that they function as classic leader cells at the wound edge (Walker et al., 2010; Bleaken et al., 2016). Interestingly, we have found that a subpopulation of the vimentin‐rich mesenchymal repair cells recruited to the leading edge in response to cataract surgery are β2 integrin, identifying them as leukocytes and demonstrating that these repair cells have immune cell properties (Fig. 1). Since this cataract surgery is performed *ex vivo*, after the lenses are isolated from the embryo, this result also provides evidence of a resident immune cell population in the lens. In lineage tracing studies, the vimentin+/CD44+ lens cell population were identified as the progenitors of the myofibroblasts associated with fibrosis in this model (Walker et al., 2018). Similarly, in human cataract surgery explants, the depletion of the mesenchymal cell subpopulation suppressed the appearance of αSMA+ cells (Walker et al., 2010; Gerhart et al., 2014). Together, these findings have opened a new avenue of study to elucidate mechanisms of wound healing and fibrosis in the lens.

### Microenvironment Determinants of Cell Fate in Response to Wounding of the Lens

The microenvironment in which wound repair takes place in the mock cataract surgery wounded explant is that of the endogenous lens basement membrane capsule. This region of the lens capsule, which is exposed when the fiber cells are extracted, is rich in basement membrane proteins typical of epithelial tissues like laminin, collagen IV, and perlecan (Danysh and Duncan, 2009). While the movement of lens epithelial cells into this matrix region for regenerative repair has been investigated, little is known about how this endogenous matrix may be modified during regenerative repair following cataract surgery wounding. In the chick *ex vivo* mock cataract surgery cultures the vimentin‐rich cells activated by repair migrate to the leading edge of the wound and direct the collective movement of the lens epithelial cells to close the wound (Menko et al., 2014a). As leader cells, the mesenchymal repair cells extend lamellipodia at the wound edge that are enriched for vimentin but surprisingly F‐actin poor (Menko et al., 2014a). Although actin stress fibers typically interact with integrin focal adhesions to mediate adhesion to the extracellular matrix and drive migration, in this leader cell population it is vimentin filaments that are link to the focal adhesions at the cells' leading edge (Menko et al., 2014a). siRNA knockdown of vimentin prevented the leader cells from extending lamellipodia and slowed wound healing, demonstrating a key role for vimentin in regulating leader cell function in wound repair (Menko et al., 2014a). In other models of wound repair, the leader cells direct the collective migration of the wounded epithelium into and across the wound. The collectivity of migration of the lens epithelium post‐wounding is provided by N‐cadherin and ZO‐1 junctions located along the cells' apical domains that are linked to a cortical actin cytoskeleton (Menko et al., 2014b). These migrating epithelial cells also are connected through a unique N‐cadherin/ZO‐1 junction that forms in the cryptic lamellipodia they extend at their forward‐moving basal domain extended toward the cell moving just in front of them, which are linked to actin stress fibers (Menko et al., 2014b). In these actively migrating epithelial cells β1 integrin has a distinct localization from N‐cadherin in the forward moving lamellipodia, similar to active myosin, which drives the forward movement of these cells across the denuded capsule (Menko et al., 2014b).

**Figure 2:**
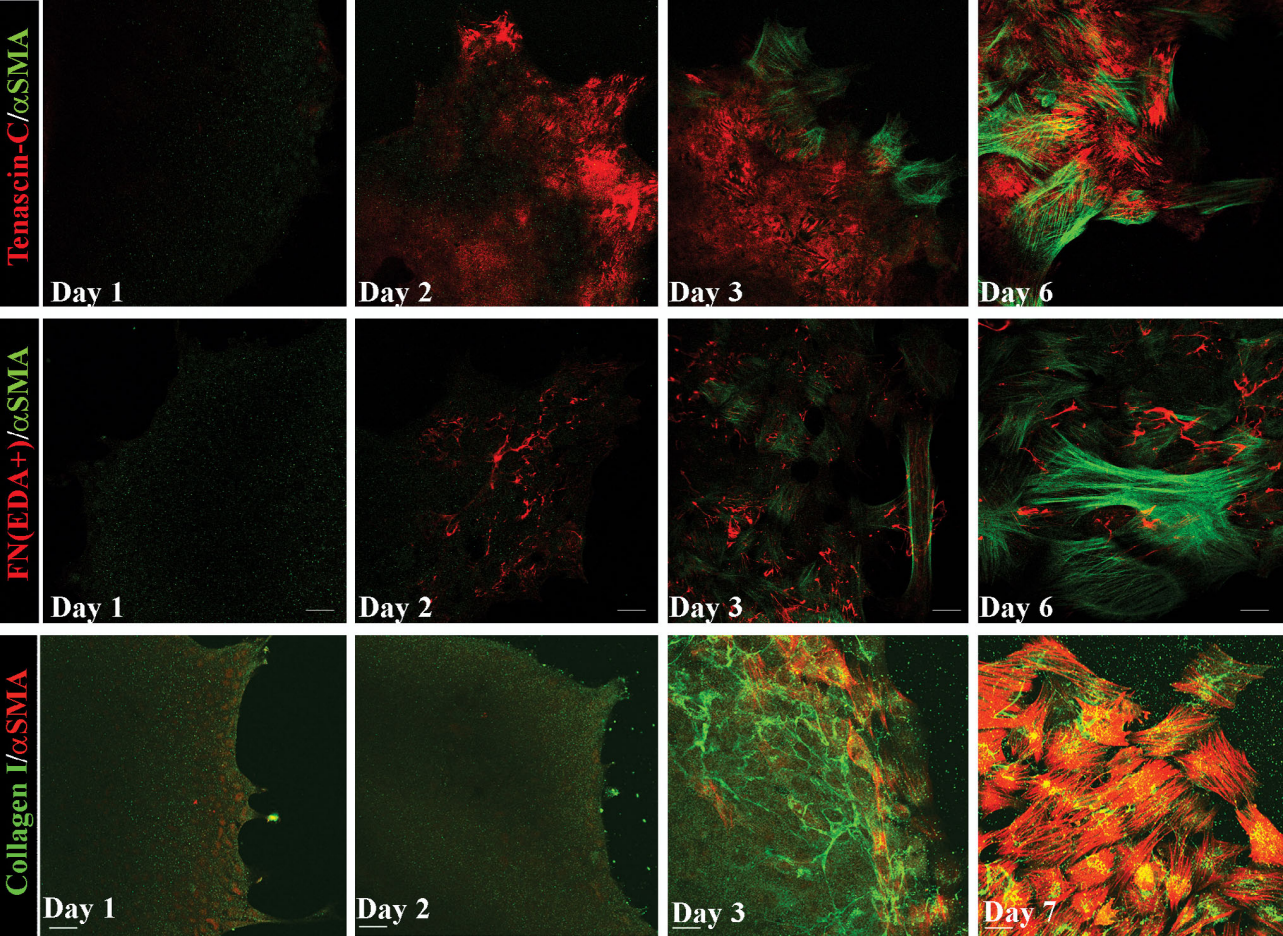
A provisional matrix forms in the mock cataract model. Following mock cataract surgery on E15 chick embryo lenses cuts are made in the anterior capsule to flatten the wounded explant on a culture platform and the lens endogenous mesenchymal repair cells migrate to this wound edge and lead the lens epithelium off the lens capsule and across the culture substrate. Explants were cultured for 6–7 days, formaldehyde fixed at D1, D2, D3, and either D6 or D7. Cells were co‐immunolabeled with antibody to α‐αSMA (Abcam) (green) and antibody to tenascin‐C (Developmental Studies) (red) (top panels), antibody to αSMA (green) and antibody to fibronectin EDA (FN(EDA+)) (Santa Cruz) (red) (middle panels), or antibody to αSMA (red) (Sigma) and antibody to collagen I (green) (ThermoFisher) (bottom panels). Fluorescent‐conjugated secondary antibodies were obtained from Jackson Immunoresearch. Images of the cells that migrated onto the culture platform were acquired with a Zeiss LSM510 confocal microscope. A provisional matrix comprises tenascin C (top panels) and fibronectin EDA (middle panels) is assembled across the substrate under the cells by culture Day 2. Collagen I fibers appear at culture Day 3 at the leading edge, produced by the mesenchymal repair cells (bottom panels), concurrent with the differentiation of these mesenchymal cells to myofibroblasts rich in αSMA.

**Figure 3:**
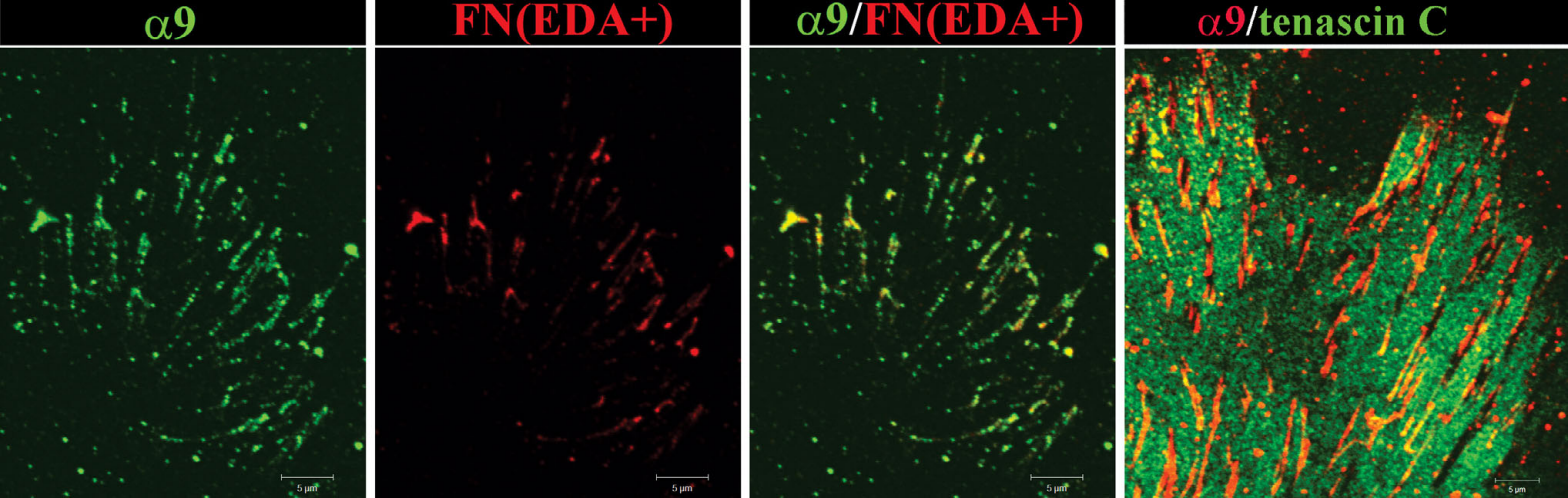
α9 integrin associates with FN‐EDA and is excluded from sites with TN‐C after mock cataract surgery. Following mock cataract surgery wounding of E15 chick lenses, co‐immunolabeling with antibodies to α9 integrin (Santa Cruz) and either fibronectin EDA (three left panels) and tenascin C (right panel) was performed to examine this integrin's interaction with the provisional matrix that is organized on the culture substrate by the cells that had migrated off of the lens capsule and across the tissue culture platform. Prior to formaldehyde fixation the cultures were treated with bis(sulfosuccinimidyl)suberate, BS^3^, a membrane impermeable amine‐to‐amine cross‐linker, to link integrins with their matrix proteins and then extracted with radioimmunoprecipitation assay (RIPA) buffer to remove all other cell proteins, making it possible to analyze only those receptors that are linked to the extracellular matrix. Cultures were imaged at the leading edge where the mesenchymal leader cell population differentiates to myofibroblasts using a Zeiss LSM510 confocal microscope. α9 integrin (green) co‐localized with fibronectin EDA (red). The tenascin C provisional matrix (red, right panel) had been removed in the region where the α9 integrin adhesion plaques had formed (red, right panel).

The collective movement of the injured lens epithelium across the surrounding tissue culture platform is similar to that which occurs in wound repair on the endogenous lens capsule, including that movement of the epithelium is directed by the subpopulation of vimentin+/CD44+ mesenchymal repair cells (Walker et al., 2018). As the cells begin to move across the tissue culture substrate they synthesize and organize a provisional matrix typical of wound repair consisting of tenascin C and fibronectin EDA (Fig. 2). The organization of these matrix proteins on the culture platform is followed by assembly of a collagen I matrix, which is produced specifically by the mesenchymal leader cells (Fig. 2). In this microenvironment, the organizational state of the leader cells' cytoskeleton is very different from when these cells locate to the wound edge created on the lens capsule. The environment of the culture platform induces the assembly of a prominent F‐actin stress‐fiber cytoskeleton, while vimentin becomes diffusely organized and localized primarily to cells' lamellipodial protrusions (Walker et al., 2018). Together, the changes in the extracellular matrix and actin organization are consistent with a mechanotransduction event that can signal the fate change of these cells to αSMA+ myofibroblasts. While it is not yet known what integrin receptor transduces this signal, one possibility is the αV integrin receptor family as cataract surgery in an αV integrin knockout mouse reduced the accumulation of fibronectin and tenascin‐C and prevented the appearance of αSMA+ cells (Mamuya et al., 2014). Our studies show that α9, a receptor for both fibronectin EDA and tenascin‐C, is enhanced at the leading edge where the repair cells acquire a myofibroblast phenotype (Fig. 3). The dependence of expression of the fibrotic molecules fibronectin and αSMA on tenascin‐C was demonstrated in lens puncture wound studies in the tenascin C null mouse (Tanaka et al., 2010).

### The Link between TGFβ, Matrix Induction, and αSMA Expression

TGFβ is a critical mediator of normal and pathological wound healing. In both wound repair and fibrosis TGFβ is a key signaling inducer of matrix proteins like fibronectin and collagen I and in fibrosis TGFβ signaling is linked to the overproduction of collagen I (Ricard‐Blum et al., 2018). Thus, an imbalance in TGFβ signaling can lead to fibrotic disease progression. In the lens, TGFβ signaling has been linked to the development of PCO (Wormstone et al., 2002; Wormstone et al., 2009; West‐Mays et al., 2010; Eldred et al., 2011; Boswell et al., 2017). The latent form of TGFβ is bound within the extracellular matrix and its activation involves integrin receptors, particularly αvβ6. Expression of this integrin is elevated when cataract surgery is performed *ex vivo* on human donor eyes (Sponer et al., 2005), and defects in TGFβ signaling are associated with an attenuation of the fibrotic response when cataract surgery is performed in αV knockout mice (Mamuya et al., 2014). Studies in the lens provide further insight into the role of the matrix in TGFβ induction of fibrosis. In human lens explants, TGFβ induction of αSMA is blocked with antibody directed against the FN Type III repeats in tenascin C (Tiwari et al., 2014). In primary cultures from human cataract surgery patients, TGFβ induction of collagen matrix production and αSMA is suppressed by exposure to function blocking antibodies to the fibronectin self‐assembly or integrin binding domains of fibronectin, which in combination have greater inhibitory effects (Tiwari et al., 2016). When TGFβ signaling is interfered with by blocking the interaction of its downstream effector Smad2/Smad4 with aldose reductase, the expressions of both fibronectin and αSMA are inhibited (Chang and Petrash, 2015). αSMA expression in TGFβ‐treated rat lens explants also can be blocked by inhibiting Rho/ROCK signaling, an activator of actin stress fiber formation (Korol et al., 2016). Advanced glycation end product posttranslational modifications of lens capsule proteins, which could increase the stiffness of the lens matrix capsule, have been correlated with increased TGFβ signaling and expression of αSMA (Raghavan et al., 2016).

While TGFβ is a potent inducer of the fibrotic phenotype, to date, the pharmaceutical efficacy of targeting TGFβ has fallen short of expectations. Other approaches to block or ameliorate PCO that impact the matrix microenvironment also hold promise. Blocking MMP2/9 function reduces αSMA expression in TGFβ‐stimulated rat lens explants (Gupta et al., 2013), and neutralizing antibody to MMP2 prevents TGFβ‐induced wrinkling of the matrix capsule in the human capsular bag model (Eldred et al., 2012). Treatment of the *ex vivo* mock cataract surgery cultures with antibodies to vimentin slowed wound healing and inhibited αSMA expression, providing the first evidence that extracellular vimentin plays a role in the development of PCO (Walker et al., 2018). It is likely that the microenvironment in which the extracellular vimentin signal is induced is a critical determinant of the outcome of this vimentin signal.

Src family kinases (SFKs) are key effectors of integrin and growth factor signaling, with functions in many pathways including cell proliferation, migration, adhesion, and the response to cell stress. Blocking SFK activation in an *ex vivo* mock cataract surgery model prevented movement of injured lens epithelium onto the posterior capsules (wound closure) and emergence of αSMA+ cells (Walker et al., 2007). Interestingly, short‐term exposure to the SFK inhibitor blocked the emergence of αSMA+ myofibroblasts without affecting cell migration (Walker et al., 2007), suggesting that blocking the activation of stress pathways could be sufficient to prevent a fibrotic response. It may also possible to prevent opacification of the posterior lens capsule post‐cataract surgery by inhibiting the wound healing response, as in the response to long‐term inhibition of SKFs. Such strategies target essential elements of the normal repair process, including lens cell proliferation, migration, and survival. One study supporting this concept used thapsigargin, which when coated on IOLs in the human capsular bag model at low concentrations reduces lens cell growth and at high concentrations induces cell death (Duncan et al., 1997). Lastly, a mechanical approach to prevent movement of cells onto the posterior capsule has been accomplished through modifications of the IOL design, and has made significant impact in the prevention of PCO (Buehl and Findl, 2008; Wormstone et al., 2009).

## LESSONS LEARNED FROM THE STUDY OF THE CORNEA

### Resident Cells Produce and Organize the Corneal Extracellular Matrix ECM into a Transparent Stroma

**Figure 4:**
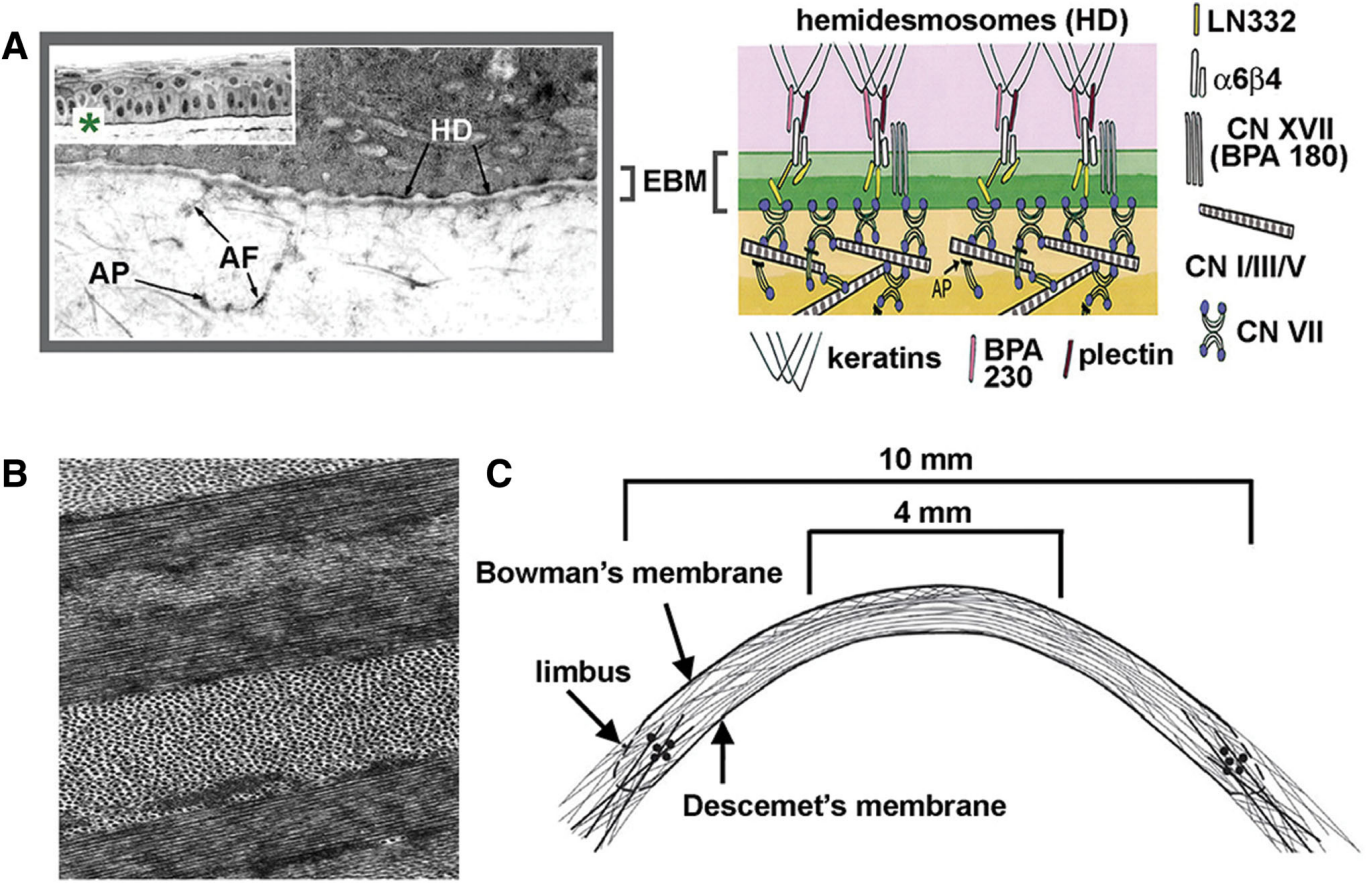
Corneal clarity depends on an intact epithelial basement membrane and a well‐ordered stromal extracellular matrix. **A**, The stratified squamous epithelium of the cornea is sheer and attached to the epithelial basement membrane (EBM) via α6β4 integrin containing hemidesmosomes (HDs) that insert into the stroma via anchoring fibers containing LN332 and adhesion plaques containing Type VII collagen (Modified and Reproduced with permission from Stepp, “The Corneal Epithelium: Cell Biology and Basic Science,” Chapter 59, in Encyclopedia of the Eye, 2010). The EBM is maintained by the epithelial cells and reassembled by them after injury. **B**, Collagen fibrils are of uniform size and spacing and are organized into sheets (lamellae) that are rotated relative to one another as shown in TEM image of the corneal stroma (Modified and Reproduced with permission from Ruberti and Zieske, Prog Retinal Eye Res, 2008, 27, 549–577. **C**, Second harmonic imaging and synchrotron X‐ray scattering were used to visualize the collagen lamellae; their organization is shown schematically. By weaving between one another, the collagen lamellae strengthen the cornea (Modified and Reproduced with permission from Abass *et al*., J R Soc Interface, 2005, 12, 20140717.

The cells that make up the quiescent stroma include keratocytes, Schwann cells, as well as resident and recruited immune cells (tissue macrophages, γδT cells, monocytes, and dendritic cells) (Hamrah et al., 2003). Although keratocytes are responsible for generating the stromal ECM during development and for maintaining it, the corneal epithelial cells and the epithelial basement membrane (EBM) that separates the epithelium from the stroma play critical roles as well. The corneal epithelium synthesizes and secretes most of the matrix proteins that assemble into the EBM (Fig. 4A; Stepp, 2010). The EBM acts as a sponge trapping cytokines and growth factors secreted by epithelial cells. The corneal endothelium also plays a role in maintaining corneal transparency. These cells regulate corneal hydration and pump nutrients from the aqueous humor delivering them to their own basement membrane (Descemet's membrane) where they diffuse into the stroma to provide nourishment to stromal and epithelial cells (Van den Bogerd et al., 2018). Corneal endothelial decompensation leads to corneal swelling and loss of transparency by disrupting the spacing between collagen fibrils.

Corneal transparency results from the precise arrangement of collagen fibrils within the stroma. The stromal keratocytes make the collagen present in the transparent stroma and organize it into fibrils and larger fibers. Integrins on their surface bind to collagen. Based on *in vitro* studies, we know that during homeostasis, keratocytes dynamically extend processes between collagen fibrils and sheets of collagen referred to as lamellae; as processes extend and retract, their integrins pull on collagen molecules exposing sites for self‐assembly and cross‐linking between fibrils to form fibers (Kadler et al., 2008; Humphrey et al., 2014; Kubow et al., 2015). Like all proteins, collagen fibrils become damaged over time and have to be removed and replaced. The major enzymes responsible for degrading collagens are MMPs. Incomplete or inadequate cleavage extracellularly coupled with impaired internalization of collagen fragments and slow lysosomal degradation intracellularly have been shown to contribute to fibrosis in the lung, liver, and skin (McKleroy et al., 2013). These data prompt scientists to suggest that fibrosis results from impaired matrix degradation and turnover rather than increased matrix production.

The primary structural components of these lamellae are collagen Type I and Type III (Cintron et al., 1981; Nakayasu et al., 1986; Power et al., 1995; Massoudi et al., 2016). While collagen Type V is present in minor amounts compared to Types I and III, it is critical for limiting the sizes of the collagen fibers that form (Birk et al., 1990; Birk, 2001). The regularity of the diameters of the fibers that form allows collagen to arrange into ordered structures within lamellae and minimize scattering of light (Fig. 4B; Ruberti and Zieske, 2008). Critical to the ordered spacing of collagen fibers relative to one another are small leucine rich repeat (SLRP) proteoglycans including decorin, lumican, fibromodulin, and biglycan (Chakravarti et al., 2000; Liu et al., 2003; Chen et al., 2014; Kamma‐Lorger et al., 2016). Studies done using mice lacking SLRPs confirmed their importance in corneal structure and transparency. Finally, non‐fibrillar collagens including collagen XII, XVII, XVIII, and XX also play critical roles in maintaining a transparent cornea (Gordon et al., 1996; Koch et al., 2001; Massoudi et al., 2016).

Advances in imaging techniques have provided insight into how collagen fibers are arranged and the relationships between lamellae that provide the cornea with its transparency and strength. Using second harmonic generation (SHG) nonlinear optical imaging to study the human corneal stroma, Winkler et al. (2013)) found sheets of collagen lamellae running transverse across the cornea which branch and intertwine with lamellae above and below as shown schematically in Figure 4C (Abass et al., 2015). Below the anterior basement membrane, collagen fibers weave in and out of Bowman's layer. Lamellar branching becomes less frequent in the posterior stroma (Meek et al., 2005; Winkler et al., 2013). The intertwining and branching of collagen lamellae provide the corneal stroma with its strength. In patients with keratoconus where the cornea thins and bulges outward, fewer collagen lamellae insert into Bowman's layer and evidence of displacement and slippage of these layers is observed by SHG imaging (Morishige et al., 2007; Morishige et al., 2009) and synchrotron X‐ray scattering (Meek et al., 2005).

### Regeneration of Transparency After Injury: Maximizing Strength While Minimizing Fibrosis

Trauma to the cornea induces repair mechanisms that lead either to regeneration and complete functional recovery or to corneal fibrosis accompanied by loss of visual acuity. The factors that determine whether a patient's cornea heals by regenerative or fibrotic repair include age, gender, overall health and nutritional status, the health of the ocular surface at the time of injury, as well as the type of trauma (Garg et al., 2005). Superficial injuries treated soon after they occur are more likely to induce regenerative repair without fibrosis. More extensive trauma that weakens the cornea by penetrating deeper into the tissue induces a stronger inflammatory and repair response generating fibrosis.

To minimize the risk of corneal rupture and rapidly restore corneal breaking strength, wound healing in the cornea evolved to occur quickly. As in the skin, a provisional matrix is first deposited onto the exposed wound site within minutes (Barker and Engler, 2017). The proteins that form the early provisional matrix (fibrin, fibronectin, and glycosaminoglycans) come from platelets and serum from leaky vessels at the injury site. In the avascular cornea, the provisional matrix is derived from the tears and from serum proteins released from blood vessels at the limbus. Resident stromal cells beneath the injured corneal epithelium in the anterior stroma disappear within minutes of injury (Wilson et al., 1996; Helena et al., 1998). Next, there is rapid resurfacing of damaged corneal epithelium to restore the barrier and allowing the removal of excess fluid in the stroma. Simultaneously, recruited and resident immune cells migrate into the wound area from the corneal periphery to begin clearing debris and the *de novo* synthesis of collagen to restore structural integrity to the cornea. Activated stromal repair cells (fibrocytes and fibroblasts) convert the early provisional matrix into a late provisional matrix composed primarily of fibronectin, collagen monomers, and glycosaminoglycans (Barker and Engler, 2017). Within this late provisional matrix, the assembly of mature collagen fibrils takes place primed by fibrillar fibronectin and collagen monomers and regulated by fibrocytes and activated fibroblasts (Kadler et al., 2008). These activated stromal repair cells pull on collagen fibrils exposing sites for cross‐linking and association between fibrils to form fibers (Kadler et al., 2008; Humphrey et al., 2014; Kubow et al., 2015). Repair cells also function like scaffolds to organize fibrils into lamellae. The more damage done to the matrix, the more proteases will be needed to degrade it and the more *de novo* collagen synthesis needed to repair the tissue. Both of these require recruitment and activation of repair cells within the stroma. From a survival perspective, the regeneration of corneal transparency after injury is secondary to restoration of corneal breaking strength. As a result, the hyperactivation of stromal repair cells and production of excess collagen will often lead to fibrosis. During the resolution phase of wound healing, immune cells recruited into the stroma in response to the injury undergo apoptosis and their contents are phagocytized by resident tissue macrophages. For optimal resolution of fibrosis, a subset of stromal repair cells is needed to continue to degrade excess collagen not assembled correctly and by synthesizing new collagen. After 6–8 months, fibrotic foci will stabilize and persist for decades. If the fibrosis is restricted to the corneal periphery and does not interfere with the tear film and blinking, it will not cause significant visual impairment. Fibrosis in the central visual axis will, however, lead to loss of vision.

Traumatic injury to the cornea is only one of several ways that corneal fibrosis can occur (Table 1, Flaxman et al., 2017). Infections are major causes of corneal fibrosis. Resident and recruited immune cells kill microorganisms; the enzymes these cells secrete often do collateral damage to the stroma by degrading collagen fibrils. If the synthesis and assembly of matrix is slower than removal of damaged collagen, the cornea will thin and eventually rupture. Collagen monomers will self‐assemble into collagen fibers and fibrils will form with varying diameters. While this strengthens the cornea, it also generates haze and/or fibrosis. After corneal strength is reestablished, stromal repair cells continue remodeling the collagen fibers in the ECM reducing haze and sizes of fibrotic areas. This process takes place over several months. To reduce blindness and visual impairment due to corneal fibrosis, efforts should focus on limiting fibrosis and extending the resolution stage to prolong remodeling of the collagen within fibrotic tissue.

**Table 1:** Causes of corneal fibrosis

Infections (bacterial, viral, and fungal) Trauma (injury, chemical, and surgery) Autoimmune disorders (Diabetes, Sjogren's Syndrome, and Epidermolysis bullosa) Corneal dystrophies (>20 subtypes recognized (Weiss et al., 2015))

Sources: http://www.who.int/blindness/en/ and Flaxman *et al*., Lancet Glob Health, 2017, 5, e1221–e1234.

Injuries to the corneal typically involve damage to or removal of the corneal epithelial cells and their EBM allowing cytokines stored in the EBM to diffuse into the stroma (Wilson et al., 2001; Torricelli et al., 2013). Wound activated corneal epithelial cells secrete growth factors and cytokines that promote stromal wound repair including TGFβ1 and TGFβ3 (Karamichos et al., 2014) that convert quiescent stromal cells into stromal repair cells. Once reepithelialization after injury is complete, the corneal epithelium reassembles the EBM and secretes collagen VII and hemidesmosomal laminin 332 into the stroma to allow stable adhesion of the epithelium to the stroma via α6β4 integrin (Stepp et al., 1990). Delays in EBM reassembly due to age or disease (corneal dystrophy, diabetes) will allow continued diffusion of cytokines from epithelial cells into the stroma and increase fibrosis (Wilson et al., 2017; Wilson et al., 2018). Stromal repair cells also secrete proteins that diffuse into the epithelium after the EBM is damaged and before it is repaired to exert paracrine effects. One such factor is hepatocyte growth factor (HGF) (Wilson, 2012; Mittal et al., 2016; Miyagi et al., 2018). In the stroma, HGF dampens fibrosis by activating Smad7 and reducing TGFβ signaling; in the epithelium, it binds to cMET and activates cell proliferation and migration. Once reassembled, the intact EBM restricts cytokine diffusion between the stroma and epithelium and signals to the corneal epithelial cells that their wound response is complete.

Immune cells are recruited into the stroma from the limbal vasculature; some monocytes will convert into fibrocytes (Lassance et al., 2018). Fibrocytes have been defined as circulating monocytes that accumulate within tissues after injury. Their functions appear similar to those carried out by resident tissue macrophages and myofibroblasts. However, studies using GFP labeled bone marrow in the mouse cornea have shown that labeled fibrocytes accumulate within the injured cornea within a day after wounding (Lassance et al., 2018); by 7 days fibrocytes begin to produce α‐smooth muscle actin (αSMA) and resemble myofibroblasts. Data show that 30%–50% of the αSMA+ cells present in the stroma 21 days after corneal injury are derived from keratocytes; the remaining αSMA+ cells are fibrocytes. Over time, both locally generated and recruited myofibroblasts undergo apoptosis and are rarely seen after wounds have resolved. Similar results have been obtained studying lung fibrosis (Misharin et al., 2017). RNAseq of resident macrophages and fibrocytes after lung injury cannot differentiate between the two cell populations.

### Cornea Fibrosis Resolution: Current Status and New Approaches to Treatment

**Figure 5:**
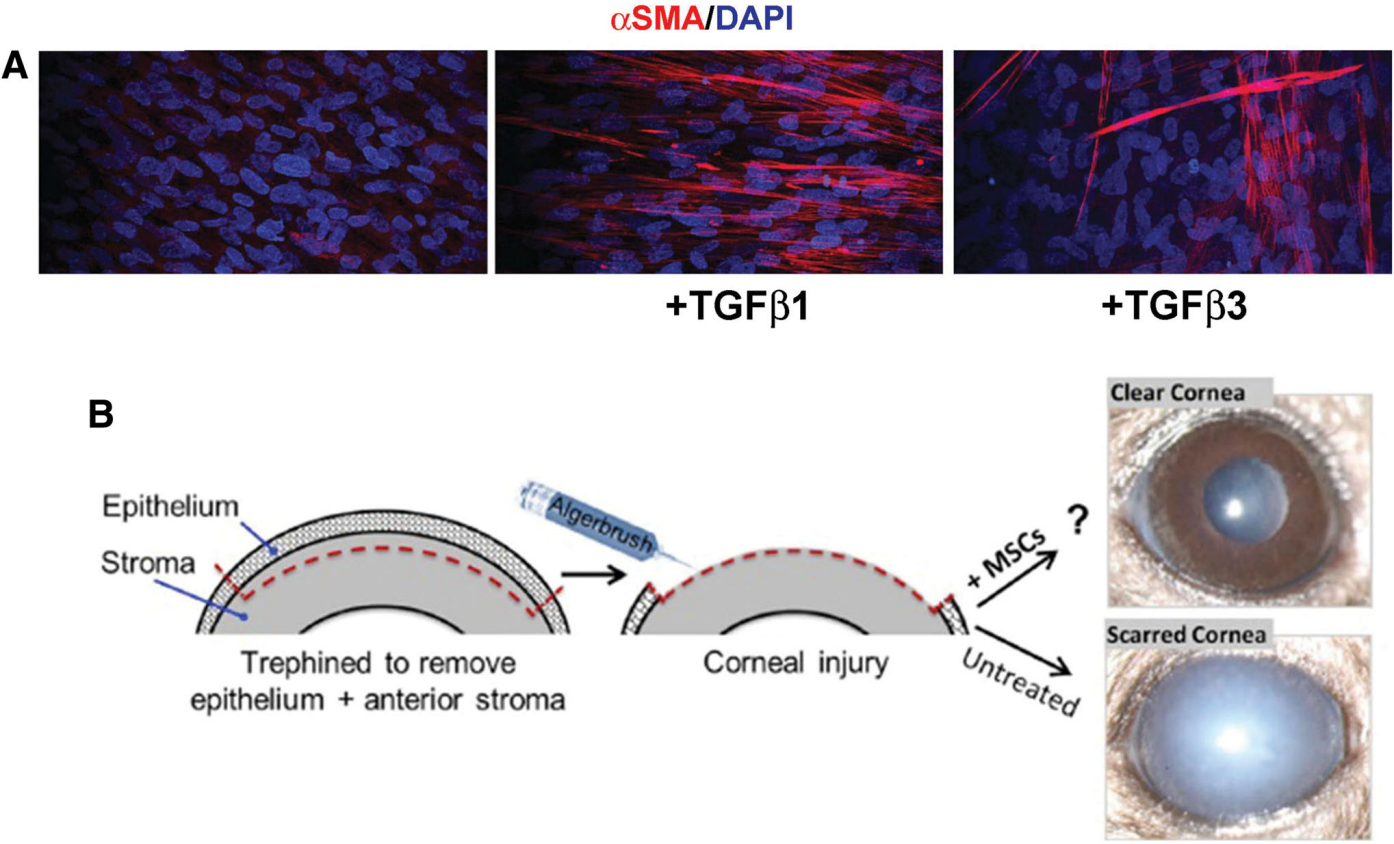
Corneal fibrosis is accompanied by expression of αSMA by corneal fibroblasts and promising new treatments to reduce fibrosis are under development using animal models of corneal injury. **A**, Corneal fibrosis is studied *in vitro* using cultures of human corneal fibroblasts. While addition of TGFβ1 to the media of cultured cells results in increased expression of αSMA, addition of TGFβ3 does not (Modified and Reproduced with permission from Sriram *et al*., Invest Ophthalmol Vis Sci, 2017, 58, 1179–1186). **B**, *In vivo* corneal injury models used to study cornea fibrosis have shown that when mesenchymal stem cells (MSCs) are injected into the stroma, corneal scarring is reduced significantly after injures that typically cause fibrosis (Modified and Reproduced with permission from Mittal *et al*., Stem Cell Rep, 2016, 7, 583–590).

Experimental models to study the formation and resolution of fibrosis in the cornea *in vivo* have given us insight into how the corneal stroma becomes disordered and ways to resolve existing fibrotic foci. These models include manual circular keratectomy, incisional, and excimer laser induced models in rodents and rabbits (Wilson et al., 2001; Stepp et al., 2014; Ljubimov and Saghizadeh, 2015; Miyagi et al., 2018). Researchers have also developed *ex vivo* models using organ‐cultured corneas to study corneal fibrosis (Karamichos et al., 2010; Karamichos et al., 2014; Chawla and Ghosh, 2018). Chemical injuries have been used to look at fibrosis (Hamill et al., 2013; Gordon et al., 2016; Baradaran‐Rafii et al., 2017; Panahi et al., 2017). Advances in cell and organ culture methodology are leading to models that are used to study both formation and resolution of fibrotic foci (Karamichos et al., 2014; Chawla and Ghosh, 2018) as shown in Figure 5A (Sriram et al., 2017).

Soon after surgery began to be used to correct refractive errors, ophthalmologists began to search for ways to reduce fibrosis at the surgical site. Mitomycin C (MMC) applied topically at the time of surgery (Santhiago et al., 2012; Majmudar et al., 2015) reduces fibrosis. Its use in the clinic has expanded to include surgeries where fibrotic wound repair would complicate recovery of function including vocal cord (Roh and Yoon, 2005) and glaucoma (Wilkins et al., 2005) surgeries. The mechanism of action of MMC on reduction of stromal fibrosis is currently being studied with a focus on the role played by the epithelial cells signaling to the stroma during wound repair (Pal‐Ghosh et al., 2016).

Many different classes of immune cells have been shown to play critical roles in mediating fibrosis in the cornea. While excess neutrophil infiltration, especially after chemical injury, contributes to increased fibrosis, elimination of neutrophils entirely delays healing (Li et al., 2006; Pal‐Ghosh et al., 2014). Neutrophils secrete cytokines which recruit other classes of immune cells at later stages of wound healing. While fibrosis in the anterior aspect of the central cornea can sometimes be removed using the excimer laser, when fibrotic foci are large and/or too deep in the stroma, the standard of care is to perform a corneal transplant (penetrating keratoplasty). The inability to obtain enough donor corneas has lead scientists to develop alternatives some of which are in use and others still under development (Garg et al., 2005). The Boston Kpro is a device that is inserted into the cornea after removal of fibrotic tissue and provides a clear visual path to allow light to hit the retina (Sayegh et al., 2008; Chew et al., 2009). Biosynthesized scaffolds containing collagen are also being investigated (Griffith et al., 2016; Ong et al., 2016; Ghezzi et al., 2017; Stern et al., 2018); fibrotic tissue would be removed and replaced with scaffolds to maintain the structure of the cornea and promote regeneration of corneal transparency via normal healing mechanisms.

A promising tool being investigated today is the use of mesenchymal stem cells (MSCs) or products derived from them to resolve existing fibrotic foci (Du et al., 2009; Liu et al., 2010; Coulson‐Thomas et al., 2013; Eslani et al., 2018). MSCs modulate the immune response when injected into injured tissues (Krampera et al., 2007; Bai et al., 2009; De Miguel et al., 2012). Studies using mouse models of corneal injury have shown that MSCs can reduce corneal fibrosis after injury (Mittal et al., 2016) (Fig. 2B). One factor secreted by MSCs is (TNF‐α)‐stimulated gene/protein 6 (TSG‐6) (Roddy et al., 2011). TSG‐6 reduces tissue fibrosis in murine full thickness skin wounds as well as fibrosis in the cornea (Qi et al., 2014). TSG‐6 modulates protease activity and innate immunity (Martin et al., 2016; Tseng, 2016) by controlling the differentiation of monocytes and fibroblasts into fibrocytes (Garlanda et al., 2005; Santhiago et al., 2011); MSCs are not the only source of TSG‐6; amniotic membranes have been used for decades to suppress inflammation of the ocular surface and enhance recovery from injury and infections (Tseng et al., 1998; Tseng et al., 1999). The characterization of TSG6 and its functions have yielded several potential new targets for development of drugs to treat fibrosis.

## CONCLUSIONS

Here we use knowledge gained using the lens and the cornea to provide insight into how fibrosis develops and clues to how it can be controlled. While the lens and cornea are less complex than other tissues that develop life‐threatening fibrosis including the lung, liver, and heart, they are well characterized and research using them as model systems to study the key elements underlying fibrosis is leading the way toward an improved understanding of fibrosis, how it is generated, and how it can be controlled to allow all tissues to regenerate fully after injury.
